# Seasonal patterns in nine notifiable communicable diseases and the epidemic dynamics of COVID-19 at Johns Hopkins Aramco Healthcare: a six-year review (2019–2024)

**DOI:** 10.25122/jml-2025-0183

**Published:** 2026-05

**Authors:** Omer kheir, Mohammad Dhalaan, Maryam Almuqrin, Razan Garwan, Fakhera Alhajri, Husam Alghamdi, Ali Al Tawfiq, Anwar AlOtaibi

**Affiliations:** 1 Population Health Department, Johns Hopkins Aramco Healthcare, Dhahran, Saudi Arabia; 2 Faculty of Public Health, London, United Kingdom; 3 Faculty of Medicine, Imam Abdulrahman Bin Faisal University, Al Khobar, Saudi Arabia; 4 Research Office, Johns Hopkins Aramco Healthcare, Dhahran, Saudi Arabia

**Keywords:** communicable diseases, seasonality, influenza, RSV, epidemiology, COVID-19, Saudi Arabia, public health

## Abstract

Seasonal variation influences the transmission dynamics of communicable diseases, yet limited evidence describes these patterns in the Eastern Province of Saudi Arabia. This retrospective study analyzed surveillance data from Johns Hopkins Aramco Healthcare (JHAH) over 6 years (2019–2024) to describe temporal and monthly patterns in nine common notifiable communicable diseases (influenza, salmonellosis, respiratory syncytial virus [RSV], chlamydia, campylobacteriosis, varicella [chickenpox], gonorrhea, scabies, and animal bites) and to describe the epidemic patterns of COVID-19 (analyzed separately from March 2020 to December 2024). The cases were organized by weekly epidemiological data and visualized through monthly line plots, illustrating temporal patterns. Descriptive statistics were used to determine annual totals, peak years and months, and case fatality rates for COVID-19. Results indicated 2,518 cases of nine notifiable diseases, predominantly influenza (686 cases), followed by salmonellosis (592), RSV (293), chlamydia (250), and campylobacter (240). Temporal trends suggestive of seasonal variations were noted, with influenza, RSV, campylobacter, and scabies peaking in winter, while salmonellosis and chickenpox peaked in October. The incidence of chlamydia, gonorrhea, and animal bites was highest in late spring and early summer. From March 2020 to December 2024, there were 36,280 COVID-19 cases and 718 deaths, with three epidemic waves and a decline in monthly cases post-2023. This exploratory study underscores the potential association of climatic and behavioral factors with disease transmission and provides preliminary observations to support public health strategies, including vaccination timing, surveillance, and further research into predictive modeling.

## Introduction

Infectious illnesses remain a major health problem worldwide, with lower respiratory infections, diarrheal diseases, and tuberculosis among the leading contributors. According to the World Health Organization (WHO) [1], lower respiratory infections, such as pneumonia and influenza, represent the leading cause of death from communicable diseases [1].

In Saudi Arabia, communicable diseases continue to strain healthcare systems, driven by respiratory infections, foodborne illnesses, sexually transmitted infections, and the lingering impacts of COVID-19 [2,3,4]. Recent national surveillance data highlight influenza, chickenpox, hepatitis viruses, salmonellosis, and brucellosis among the most commonly reported conditions, with notable disruptions and resurgences post-COVID-19 [3,4]. Climate change exacerbates these challenges in Gulf Cooperation Council (GCC) countries, including Saudi Arabia, through rising temperatures, extreme weather events, and altered transmission dynamics for vector-borne and respiratory pathogens [5].

A retrospective analysis studied how seasons affect the prevalence of infectious diseases in Riyadh Province. The research found that microbial infections increased during certain months: bacterial infections peaked in June through September, viral infections were most common from December to March, and fungal infections increased in July and August. No clear seasonal pattern was observed for protozoan infections [6]. The analysis also showed a strong association between warmer months and bacterial infections, and between cooler months and viral infections, indicating that different pathogens tend to be more active at different times of the year [6].

Several studies have documented the most frequently reported diseases in Saudi Arabia, their trends over the years, and their implications for surveillance. One study analyzed Ministry of Health data from the Field Epidemiology Training Program (FETP) in Saudi Arabia for 2018-2021. The findings of this study show that the most reported viral diseases included hepatitis B, hepatitis C, dengue fever, influenza, chickenpox, and measles [3]. Another study examined bacterial diseases in the same period using Saudi epidemiology surveillance data. The study revealed that brucellosis, tuberculosis (both pulmonary and extrapulmonary forms), and salmonellosis were the most commonly reported diseases [4].

Seasonal patterns have been documented in multiple studies, including research conducted at King Abdulaziz Medical City, especially for respiratory viruses such as influenza, respiratory syncytial virus (RSV), adenovirus, and rhinovirus/enterovirus. These studies highlight important seasonal patterns and age-based occurrence of respiratory infections, showing increases during the winter months, especially for rhinovirus/enterovirus, SARS-CoV-2, RSV, and adenovirus [7]. Another study conducted between 2015 and 2022 at King Fahd Hospital of the University (KFHU) in Saudi Arabia’s Eastern Province showed that RSV infections were more frequent from August to February, with a noticeable drop occurring from March to July [8]. Both studies revealed that respiratory viruses are most commonly seen in children younger than 5 years, although they can also infect older individuals [7,8].

A comprehensive review identified the Jazan region as particularly vulnerable to communicable and vector-borne diseases, owing to its geographic, socioeconomic, and climatic features. The study analyzed data from 2006 to 2021 and found that malaria, hepatitis B, dengue fever, chickenpox, and pulmonary tuberculosis were the most commonly reported notifiable communicable diseases in the region [9]. In the southwest part of Saudi Arabia, a research project examined viral respiratory illnesses in children caused by RSV, SARS-CoV-2, and influenza. Researchers observed seasonal variations, with RSV infections rising during specific months, highlighting the need for further studies to fully understand these trends [2].

Prior to the study period, commonly reported communicable diseases at Johns Hopkins Aramco Healthcare (JHAH) included mumps, influenza, chickenpox, COVID-19, gonorrhea, scabies, salmonellosis, RSV, chlamydia, and campylobacteriosis, among others. For this analysis, we focused on the nine notifiable diseases with sufficient cases for robust seasonal assessment (influenza, salmonellosis, RSV, chlamydia, campylobacteriosis, varicella, gonorrhea, scabies, and animal bites), with COVID-19 examined separately due to its distinct epidemic nature.

This retrospective study addresses the gap in localized temporal data for the Eastern Province by analyzing 6 years (2019–2024) of surveillance records from JHAH. By exploring temporal patterns and their potential associations with climatic and behavioral factors, these findings may support the development of targeted public health interventions, such as optimized vaccination timing, enhanced surveillance, and resource allocation in similar arid settings.

## Material and Methods

### Design and study setting

This retrospective cohort study was conducted from January 2019 to December 2024. Data were collected from EPIC, an electronic health record system, at JHAH in the Eastern Province of Saudi Arabia. JHAH provides comprehensive medical coverage for Aramco employees and their eligible dependents. The coverage extends across all JHAH facilities, including Dhahran, Al-Hasa, Ras Tanura, Abqaiq, and Udhallyah.

### Data source and collection

Data were extracted from the JHAH electronic health record system (EPIC). All laboratory-confirmed or clinically diagnosed cases of notifiable communicable diseases, as defined by the Saudi Ministry of Health, were included. The nine diseases selected for seasonal analysis were influenza, salmonellosis, respiratory syncytial virus (RSV), chlamydia, campylobacteriosis, varicella (chickenpox), gonorrhea, scabies, and animal bites (included due to rabies transmission risk and notifiable status). COVID-19 cases were analyzed separately from March 2020 to December 2024 due to their distinct epidemic pattern. Patient demographics, diagnosis codes, dates of diagnosis, and facility locations were extracted. All personal identifiers were removed prior to analysis to ensure confidentiality.

### Sampling, inclusion, and exclusion criteria

All confirmed cases of reportable diseases, as defined by the Saudi Ministry of Health, with positive laboratory results at JHAH, encompassing all age groups, were included in the study. However, people outside the scope of the JHAH system, duplicate, and incomplete records were excluded.

**Data analysis**

Cases of the nine notifiable diseases were aggregated by Sunday-based epidemiological weeks, according to the Centers for Disease Control and Prevention / Morbidity and Mortality Weekly Report (CDC/MMWR) standard, from epidemiological week 1 of 2019 to week 52 of 2024. For temporal visualization, each epidemiological week was assigned to its corresponding Gregorian calendar month based on the week’s starting Sunday. COVID-19 cases were aggregated by calendar month of diagnosis.

Monthly aggregation was chosen to facilitate consistent cross-year comparison of temporal patterns and to reduce the impact of short-term fluctuations (e.g., holiday or reporting delays) while still capturing biologically plausible temporal patterns. This approach smooths intra-month variation and may obscure the exact timing of short outbreaks or peaks within a month; therefore, observed patterns should be interpreted as representative of monthly-level trends rather than precise weekly dynamics.

Descriptive statistics included total and annual case counts, mean, median, and maximum annual cases, peak year (calendar year with the highest cases), and peak month (calendar month with the highest cumulative cases across all years; earliest month selected in case of ties). For each infection, a simple numerical summary of the mean and median monthly cases (along with minimum, maximum, standard deviation, and the proportion of zero-case months) across the 6-year period was calculated and reported in Supplementary Table 1 to support the interpretation of the consistency and strength of the seasonal patterns observed in the line plots. Case fatality rates (CFR) were computed for COVID-19 (deaths ÷ confirmed cases × 100).

Supplementary File

**Table 1:** Summary of cases for nine notifiable communicable diseases at JHAH, 2019–2024

Disease	Total cases (2019 – 2024)	Mean annual cases	Median annual cases	Max annual cases	Year of peak	Peak month
INFLUENZA	686	114	112	238	2023	Nov
SALMONELLOSIS	592	99	98	133	2019	Oct
RSV	293	49	40	110	2024	Nov
CHLAMYDIA	250	42	40	53	2022	Jun
CAMPYLOBACTER	240	40	36	67	2024	Dec
SCABIES	225	38	36	53	2020	Dec
CHICKEN POX	197	33	32	48	2019	Oct
GONORRHEA	159	26	29	39	2022	May
ANIMAL BITE^1^	76	13	14	19	2019	May

Animal bites were included as a notifiable category due to potential rabies transmission risk, per Saudi Ministry of Health guidelines. Peak month = the month with the highest count in that year

Temporal patterns were visualized using monthly line plots (one line per year, 2019–2024) with meteorological season shading: winter (December–February, light blue), spring (March–May, light green), summer (June–August, light yellow), and autumn (September–November, light orange). COVID-19 was plotted as a continuous monthly time series with the same seasonal shading.

All data processing, calculations, and visualizations were performed using R statistical software version 4.4.1 (packages: dplyr, ggplot2, lubridate).

## Results

### Notifiable diseases (2019-2024)

A total of 2,518 laboratory-confirmed or clinically diagnosed cases of the nine selected notifiable communicable diseases were reported at Johns Hopkins Aramco Healthcare from 2019 to 2024 (Table 1). Influenza was the most frequent (*n* = 686; 27.2%), followed by salmonellosis (*n* = 592; 23.5%), respiratory syncytial virus (*n* = 293; 11.6%), chlamydia (*n* = 250; 9.9%), and campylobacteriosis (*n* = 240; 9.5%). The remaining diseases—varicella (chickenpox), gonorrhea, scabies, and animal bites—accounted for the remaining cases, with animal bites contributing the fewest (*n* = 76; 3.0%).

Annual case totals varied, with the highest single-year count for influenza occurring in 2023 (238 cases). Peak years differed by pathogen: salmonellosis, varicella, and animal bites peaked in 2019; scabies in 2020; chlamydia and gonorrhea in 2022; and influenza, RSV, and campylobacteriosis in 2023 or 2024.

**Figure 1:**
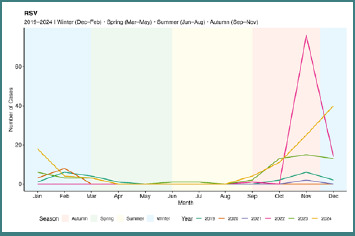
Temporal pattern of RSV

**Figure 2:**
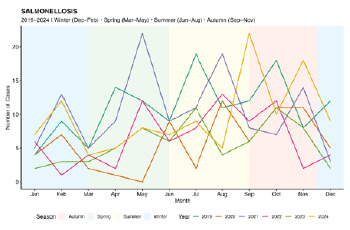
Temporal pattern of salmonellosis

**Figure 3:**
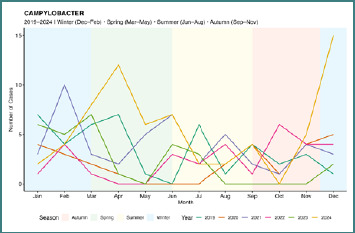
Temporal pattern of Campylobacter

**Figure 4:**
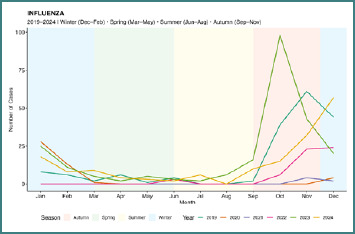
Temporal pattern of influenza

**Figure 5:**
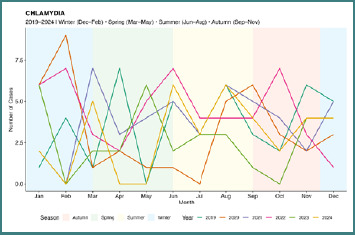
Temporal pattern of chlamydia

**Figure 6:**
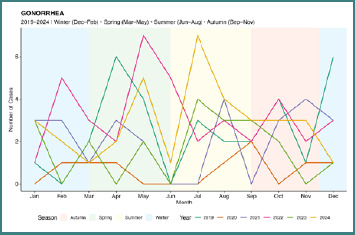
Temporal pattern of gonorrhea

**Figure 7:**
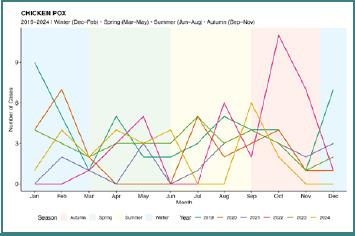
Temporal pattern of chicken pox

**Figure 8:**
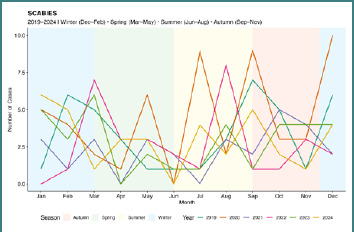
Temporal pattern of scabies

**Figure 9:**
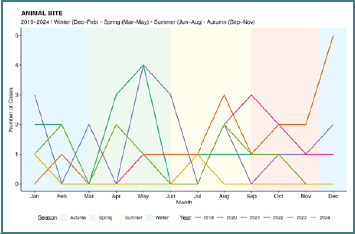
Temporal pattern of animal bite

Distinct temporal patterns were observed across the nine diseases (Figures 1–9). Influenza, RSV, campylobacteriosis, and scabies showed apparent winter predominance, with peak months in November or December. Salmonellosis and varicella peaked in October (autumn). Chlamydia, gonorrhea, and animal bites reached their highest incidence in late spring or early summer (peak months: June for chlamydia, May for gonorrhea, and May for animal bites). To quantify the consistency of temporal patterns observed in Figures 1–9, see Supplementary Table 1 for summary statistics of monthly case counts (mean, median, standard deviation, minimum, maximum, and proportion of zero-case months) for each infection across the 6-year period.

### COVID-19 (March 2020-December 2024)

**Figure 10:**
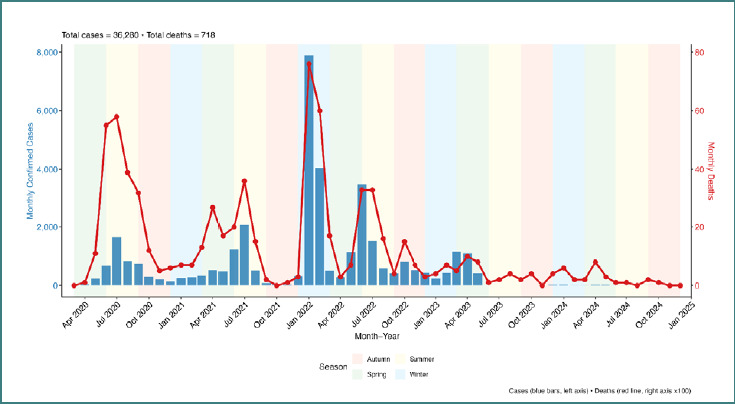
COVID-19 cases and deaths

Separately, 36,280 laboratory-confirmed COVID-19 cases were recorded at JHAH from March 2020 through December 2024 (Table 2; Figure 10). The epidemic comprised three major waves: the first peaked in July 2020 (highest mortality, *n* = 219 deaths), the second in July 2021 (2,074 cases), and the largest Omicron-driven wave peaked in January 2022 (highest case count). A smaller resurgence occurred in early 2023. Case counts subsequently declined sharply, remaining below 30 per month throughout 2024. A total of 718 deaths were reported over the period (overall CFR 1.98%), with the highest absolute mortality occurring during the initial wave in 2020 (CFR 4.49%) and a notably elevated CFR of 17.45% in 2024 due to the very low volume of residual cases.

**Table 2:** Annual COVID-19 cases and deaths at JHAH, March 2020 – December 2024

Year	Confirmed cases	Deaths	Case fatality rate (%)	Peak month (Cases)	Peak month (Deaths)
**2020**	4,874	219	4.49	Jul	Jul
**2021**	6,196	148	2.39	Jul	Jul
**2022**	21,663	274	1.26	Jan	Jan
**2023**	3,398	51	1.5	Mar	Apr
**2024**	149	26	17.45	Jan	Apr
**Total (Mar 2020 – Dec 2024)**	36,280	718	1.98	Jan 2022	Jul 2020

CFR = (Deaths ÷ Confirmed Cases) × 100. Peak month = the month with the highest count in that year

## Discussion

Over the six-year surveillance period at JHAH, influenza consistently ranked as the most frequently reported communicable disease. Salmonellosis was the second most common, followed by respiratory syncytial virus, chlamydia, and Campylobacter. This distribution reflects a diverse burden of disease in the community, encompassing respiratory infections, foodborne illnesses, and sexually transmitted infections (STIs). While the overall numbers went up and down from year to year, each disease had its own pattern—some peaked early, others showed up more recently. Temporal patterns were notably evident: influenza, RSV, campylobacter, and scabies showed distinct winter dominance, correlating with lower temperatures and the seasonal increase in respiratory and close-contact illnesses. Conversely, salmonellosis and chickenpox reached their highest levels in the fall months, suggesting associations with food preparation practices and seasonal shifts. Chlamydia, gonorrhea, and animal bites were prevalent during late spring and early summer, indicating behavioral and environmental influences tied to warmer temperatures [4].

COVID-19 followed a different path, characterized by three major epidemic waves from 2020 to 2022, after which case numbers decreased dramatically and remained low until the end of the study period. Collectively, these results highlight steady seasonal patterns and evolving epidemic dynamics across various disease types.

The prevalence of influenza and RSV in winter is influenced by weather changes, as indoor environments and low humidity promote their transmission [7]. The increased number of influenza cases in 2023 is consistent with worldwide reports of a recovery following the pandemic, driven by changes in population immunity [2]. The peak of RSV cases in 2022 also aligns with earlier data showing that its seasonal aggregation occurs between August and February in Saudi Arabia [7].

Likewise, the peak month for animal bites was May, indicating more time spent outside during warmer weather. The winter increase in scabies and Campylobacter suggests that cold weather and seasonal behavior changes might increase indoor transmission or food contamination risks [4]. Salmonellosis showed a distinct trend in the fall, peaking in October. This corresponds with earlier Saudi studies showing increased instances of bacterial infections during the shift from warm to cooler months [4].

A study showed a clear autumn peak in foodborne diseases, including salmonellosis and campylobacteriosis [10]. This aligns with global evidence implicating warmer weather and food-handling practices as potential drivers of bacterial transmission [11]. Exacerbating this might be behavioral factors that encourage travel, the consumption of particular foods, and outdoor activities. Other studies have shown that temperature changes during transitional seasons can amplify the growth of these foodborne pathogens [12].

The COVID-19 data in our study showed three distinct waves of the epidemic, which correspond to the global emergence of new variants. The highest number of deaths occurred during the first wave in 2020 (CFR 4.49%), while the highest monthly case count occurred during the Omicron wave in January 2022. Severity is not the reason behind the significantly elevated CFR in 2024 (17.45%). It is influenced by individual fatalities from a limited number of cases [13]. Considering that RSV and influenza cases increase in the wintertime, starting vaccination campaigns earlier, ideally in September or October, and allocating resources for hospital emergency rooms throughout the winter are recommended [2].

Comparable trends have been observed throughout the United States and Europe, where respiratory viruses decreased significantly during COVID-19 restriction phases and increased again after non-pharmaceutical measures were removed [14,15]. The significant return of influenza in 2023, as reflected in our dataset, also aligns with recent global studies indicating delayed or increased influenza outbreaks during the recovery phase [16].

Taken together, the alignment between our results and the established literature strengthens the validity of the observed seasonal patterns and underscores the significant impact of climate, behavior, and population movements on infectious disease dynamics. It highlights the need for targeted seasonal interventions, immunization strategies, and early warning systems based on regional surveillance and global epidemiological knowledge.

Given that salmonella and chickenpox cases increase in the autumn season, this highlights the need for food safety campaigns and prevention measures, especially in September and October. Furthermore, the peak in sexually transmitted infections and animal bites in spring and early summer indicates that well-planned health education is most effective during these seasons. The lessons learned from the various COVID-19 waves emphasize the importance of rapidly increasing capacity when necessary. The reduction in cases after the beginning of 2023 suggests sustained, widespread immunity; however, the initially high mortality rate underscores the importance of a rapid response when new outbreaks occur. Collectively, these findings support tailored interventions that leverage seasonal trends, appropriate resource allocation, and enhanced understanding among healthcare providers and policymakers.

A key limitation of this study is the impact of the COVID-19 pandemic, which significantly altered how individuals accessed medical care, conducted testing, and reported clinical outcomes between 2020 and 2021.

These disruptions may have significantly diminished or amplified the observable trends of other communicable diseases, complicating the assessment of whether the variations noted were genuine shifts in disease patterns or merely indicative of altered behaviors during the pandemic.

Lockdown measures likely reduced reporting, particularly of mild or self-limiting cases, so observed variations during this period may reflect reporting bias rather than true changes in disease incidence.

Additionally, the study’s reliance on data from a single healthcare system may limit the generalizability of its findings to the broader Saudi Arabian population. The retrospective design depends on the accuracy and completeness of existing clinical records, introducing the possibility of information bias. Surveillance data may not adequately capture mild or unreported cases, as individuals who did not seek medical care would be excluded. Furthermore, the absence of detailed demographic breakdowns, such as age and sex, limits the ability to assess population-specific patterns and risk differences.

Despite these limitations, the study has several strengths, such as a comprehensive multi-year dataset, standardized case reporting, and thorough coverage of various disease categories. These strengths can help public health officials devise more effective prevention strategies by providing valuable insights into long-term seasonal trends.

Given seasonal peaks in respiratory viral infections, particularly during October–December, vaccination campaigns for influenza and RSV should be initiated earlier, ideally in September, to ensure adequate population immunity before peak transmission. Aligning vaccination timing with these documented seasonal patterns may enhance vaccine effectiveness and reduce disease burden during high-risk months [17].

More generally, future studies should use data from multiple centers or nationwide sources to make the results more applicable to the wider population. Improving disease surveillance by better data collection, more complete reporting, and the inclusion of basic demographic information, such as age and sex, would enable clearer analysis of disease patterns. Using stronger analytical methods may also help predict disease trends and support timely public health decisions.

## Conclusion

This six-year exploratory descriptive analysis of notifiable communicable diseases at Johns Hopkins Aramco Healthcare provides evidence of temporal patterns consistent with seasonal variation in disease occurrence in the Eastern Province of Saudi Arabia. Distinct temporal patterns were observed, with influenza, RSV, campylobacter, and scabies demonstrating apparent winter predominance; salmonellosis and chickenpox peaking in the autumn; and chlamydia, gonorrhea, and animal bites showing higher incidence during late spring and early summer. These trends suggest potential roles for climate, population behavior, and environmental conditions in shaping disease transmission dynamics. The waves of the COVID-19 pandemic—which had very noticeable peaks in 2020, 2021, and 2022—further underscore the need for flexible public health preparedness and rapid response systems. Even though the number of COVID-19 cases decreased significantly after the start of 2023, the pandemic offers important lessons about monitoring for diseases, detecting them early, and preparing resources in advance. Overall, the findings of this study provide actionable insights for optimizing vaccination schedules, anticipating healthcare demand, strengthening public health messaging, and planning preventive interventions tailored to seasonal risk periods. Continued research that integrates climatic, demographic, and behavioral factors will enhance understanding of disease drivers and support the development of predictive tools for early warning and preparedness. By identifying consistent seasonal trends, this study contributes to more efficient, data-driven public health strategies to reduce the burden of communicable diseases in the region.

## Data Availability

The data are not publicly available because they contain information that could compromise patient privacy. De-identified data may be available from the corresponding author upon reasonable request and with institutional approval.
