# Supplementary material for: Seasonal patterns in nine notifiable communicable diseases and the epidemic dynamics of COVID-19 at Johns Hopkins Aramco Healthcare: a six-year review (2019–2024)
Source: J Med Life. 2026 May;19(5):366–72. doi: 10.25122/jml-2025-0183 (PMC13389803; doi:10.25122/jml-2025-0183)
Supplement: Supplementary file 1 [file JMedLife-19-366-s001.pdf]

Supplementary Table 1. Monthly case summary by infection (2019-2024) aggregated across all calendar months and years

Infection	Mean monthly cases	Median monthly cases	Min monthly cases	Max monthly cases	Months with zero cases	% Months with zero cases
Influenza	9.5	2.0	0	98	29	40.3
Salmonellosis	8.2	8.0	0	22	1	1.4
RSV	4.0	0.0	0	76	43	59.7
Chlamydia	3.5	3.0	0	9	8	11.1
Campylobacter	3.3	3.0	0	15	12	16.7
Scabies	3.1	3.0	0	10	6	8.3
Chickenpox	2.7	3.0	0	11	14	19.4
Gonorrhea	2.2	2.0	0	7	14	19.4
Animal Bite	1.1	1.0	0	5	29	40.3

Statistics are based on calendar-month aggregation of Sunday-based epidemiological weeks. Months with zero cases reflect the true absence of reported cases in that month across the study period.

© 2026 by the authors. This article is an open access article distributed under the terms and conditions of the Creative Commons Attribution (CC BY 4.0) license.
